# Robotic versus laparoscopic anti-reflux and hiatal surgery in a uk benign upper gi unit: perioperative outcomes in a four-year observational study

**DOI:** 10.1007/s11701-026-03988-0

**Published:** 2026-09-21

**Authors:** Jada Saunders, Mustafa Al-Qasem, Edward J Nevins, Alexander Green, Sadiq Bawa, Liam Horgan

**Affiliations:** 1 Department of Upper GI Surgery, Northumbria Healthcare NHS Foundation Trust, Cramlington, United Kingdom; 2 Northern Surgical Trainee Research Association (NOSTRA), Newcastle upon Tyne, United Kingdom

**Keywords:** Fundoplication, Robotic, Laparoscopy, Reflux, Hernia

## Abstract

Robotic fundoplication is rapidly expanding across the UK, however, robust comparative data from routine benign upper gastrointestinal (UGI) practice remain limited. This study compares perioperative outcomes of robotic and laparoscopic fundoplication within a UK benign UGI unit. This retrospective observational study included consecutive adults undergoing elective laparoscopic or robotic anti-reflux or hiatal surgery between February 2022 and January 2026. The primary outcome was total operative time. Secondary outcomes included length of stay, same-day discharge, complications, 30-day readmission, 30-day return to theatre, subsequent redofundoplication and early clinically recorded recurrence. One hundred patients were included: 57 robotic and 43 laparoscopic. Revisional surgery was more frequent robotically (33.3% versus 11.6%; *p* = 0.006). Overall, robotic procedures had shorter median operative times (88 versus 120 min; *p* = 0.017) and higher same-day discharge rates (63.2% versus 27.9%). After excluding the highest-volume robotic surgeon, median robotic operative time remained 27 min shorter than laparoscopy (93 versus 120 min), although the difference was no longer statistically significant (*p* = 0.16). Among 76 primary operations, robotics was associated with shorter unadjusted operative time (77 versus 120 min; *p* < 0.001), shorter length of stay (0 versus 1 day; *p* = 0.003) and higher same-day discharge (60.5% versus 23.7%; OR 4.94, 95% CI 1.83–13.31; *p* = 0.002). Following covariate adjustment, operative time remained an estimated 11% shorter robotically, although the confidence interval included no difference (time ratio 0.89, 95% CI 0.72–1.09; *p* = 0.250). The association with same-day discharge persisted after adjustment (OR 5.02, 95% CI 1.09–23.00; *p* = 0.038). Robotic anti-reflux and hiatal surgery was successfully integrated into an established benign UGI service, including for a substantial revisional workload. Favourable operative times and same-day discharge rates demonstrate the potential for efficient service delivery, although the respective contributions of platform, surgeon expertise, case selection and treatment era cannot be separated. These findings demonstrate feasibility and support further prospective evaluation of its comparative clinical and economic value.

**Supplementary Information:**

The online version contains supplementary material available at https://doi.org/10.1007/s11701-026-03988-0.

## Introduction

 Following its first description in 1991 [1], laparoscopic fundoplication was rapidly adopted internationally and became established as the standard minimally invasive approach to anti-reflux surgery. Compared with open surgery, laparoscopy offered reduced postoperative pain, shorter hospital stay and faster functional recovery, fundamentally transforming perioperative outcomes in foregut surgery. Open fundoplication is now uncommon in contemporary UK practice, with more than 99% of elective fundoplications performed using minimally invasive techniques [2]. Building upon this minimally invasive foundation, robotic platforms represent the latest evolution in the delivery of anti-reflux and hiatal surgery.

Robotic-assisted fundoplication is increasingly used as an alternative to conventional laparoscopy [3–6]. Association of Upper Gastrointestinal Surgery of Great Britain and Ireland (AUGIS) consensus work has highlighted the rapid expansion of robotic surgery, reflected in NHS administrative data showing an increase in recorded robotic procedures from approximately 3,401 in 2012/13 to 37,751 in 2022/23 [7]. Robotic platforms provide stable three-dimensional visualization, articulated instruments and improved ergonomics, which may be advantageous during hiatal dissection, crural suturing and revisional surgery. However, these technical capabilities do not necessarily translate into superior patient-level outcomes. Published UK experience in hiatal and anti-reflux surgery remains limited and is predominantly derived from tertiary referral centres and high-volume institutions with established robotic infrastructure [6]. Evidence regarding the implementation of robotic surgery within routine benign upper gastrointestinal services therefore remains limited.

Appropriate patient selection and perioperative assessment remain important in anti-reflux and hiatal surgery. Perioperative assessment was informed by the Audit and Review of Anti-Reflux Operations and Workup (ARROW) framework and relevant AUGIS, British Society of Gastroenterology and International Consensus on Anti-Reflux Surgery recommendations, recognising that investigation requirements vary according to surgical indication [2, 8–10]. Preoperative investigations are therefore reported descriptively to provide clinical context rather than as a uniform measure of guideline adherence across this heterogeneous cohort.

This study presents a consecutive four-year institutional experience of laparoscopic and robotic anti-reflux and hiatal surgery within a UK benign upper gastrointestinal service. It evaluates operative delivery and early postoperative outcomes, with primary-only, adjusted and sensitivity analyses used to explore the influence of case mix, surgeon concentration, operative technique and treatment era. The study aims to assess the feasibility of integrating robotic hiatal and anti-reflux surgery into routine benign upper gastrointestinal practice.

### Aim

This study aimed to evaluate the feasibility, implementation and perioperative performance of robotic anti-reflux and hiatal surgery within an established benign upper gastrointestinal service, using laparoscopic surgery as a contemporary comparator. The primary outcome was total operative time. Secondary outcomes included length of stay, same-day discharge, complications, 30-day readmission, 30-day return to theatre, subsequent redo fundoplication and early clinically recorded recurrence.

## Methods

Data were recorded prospectively through intraoperative logs and electronic patient records and analysed retrospectively. A search for “fundoplication” was performed using the institutional electronic theatre database (SIRIS). Adults undergoing elective laparoscopic or robotic anti-reflux or hiatal surgery between February 2022 and January 2026 at Northumbria Healthcare NHS Foundation Trust were included. Eligible procedures comprised primary anti-reflux surgery, primary anatomical hiatal or paraoesophageal hernia repair, and revisional surgery following previous anti-reflux or hiatal intervention. Procedures incorporating gastropexy were included. Emergency procedures and operations performed in conjunction with Heller myotomy were excluded, as outcomes following Heller myotomy have been reported separately [11]. The study was conducted in accordance with STROBE reporting guidelines [12]. As a retrospective service evaluation, formal ethical approval was not required.

The Trust is a secondary-care NHS provider serving a mixed urban and rural population and delivering a dedicated benign upper gastrointestinal service, including referrals from neighbouring centres. The service encompasses benign biliary surgery, complex abdominal wall reconstruction, hiatal and anti-reflux surgery, bariatric surgery, and integrated diagnostic support including in-house endoscopy and oesophageal physiology testing. Eight consultants contributed to the study cohorts. Four of them, were formally credentialled in robotic anti-reflux and hiatal surgery and performed all robotic procedures during the study period; these surgeons also undertook some laparoscopic procedures. Robotic credentialling was achieved through structured proctorship and institutional approval before independent practice. Bedside assistance was provided by a robotic-trained surgical care practitioner who had completed formal platform training. Laparoscopic procedures were predominantly performed by consultants, with selected cases undertaken by surgical trainees under direct consultant supervision. All participating consultants were experienced upper gastrointestinal surgeons. With the exception of one surgeon contributing to the laparoscopic cohort, each had more than 10 years of independent surgical experience. The highest-volume robotic surgeon had extensive laparoscopic foregut experience but commenced robotic fundoplication during the study period after structured proctorship and institutional credentialling.

Operative approach was determined during preoperative planning by the operating consultant according to clinical judgement, procedural complexity, previous surgery, surgeon experience and robotic availability. There were no formal allocation criteria or randomization. Robotic procedures were performed using the da Vinci Xi platform with a standard four-arm docking approach. Patients were positioned in reverse Trendelenburg with their arms by their sides. Initial access was obtained through a 12-mm umbilical entry, followed by placement of three 8-mm robotic ports. A Nathanson liver retractor was used for left-lobe retraction. The robotic setup typically included Cadière forceps in the lateral working ports and a monopolar diathermy hook in the medial port for dissection. Advanced disposable energy devices were not routinely used in robotic cases. Laparoscopic procedures were performed in a similar reverse Trendelenburg position using a four-port technique. A 12-mm umbilical camera port was placed, with one 10-mm working port in the right upper quadrant and two 5-mm ports in the left upper quadrant. A Nathanson liver retractor was used in all cases. Dissection was performed using an energy device, with atraumatic graspers used for gastric retraction.

Patients considered suitable for day-case surgery could be identified preoperatively following anaesthetic assessment, although same-day discharge was not guaranteed in advance. The final decision was made following postoperative clinical assessment. Discharge criteria included physiological stability, tolerance of oral fluids at four hours and oral medication, adequate pain and nausea control, mobilisation, passage of urine and appropriate home support.

Data were collected in alignment with the ARROW audit framework (Supp. Table S1) [2]. Recorded patient characteristics included age, sex, body mass index and ASA grade. Operative variables included operation date, surgical approach, total operative time, indication, primary or revisional status, operating consultant, trainee participation, wrap type and crural repair technique. Docking and console times were recorded for robotic procedures. Preoperative investigations included gastroscopy, oesophageal manometry, pH testing and barium swallow. These were reported descriptively because the indications for physiological testing differed between primary reflux surgery, predominantly anatomical hiatal or paraoesophageal hernia surgery, and revisional procedures. Postoperative outcomes included length of stay, same-day discharge, complications graded according to the Clavien–Dindo classification, 30-day readmission, 30-day return to theatre, subsequent redo fundoplication and clinically recorded hiatal hernia recurrence. Follow-up variables included provision of dietary advice, proton pump inhibitor status at the latest documented review, follow-up provider, discharge from surgical follow-up and documented follow-up duration.

Revisional surgery was defined as an index procedure performed in a patient who had previously undergone anti-reflux or hiatal surgery. Return to theatre was defined as an unplanned reoperation within 30 days of the index procedure. Redo fundoplication referred to a subsequent repeat anti-reflux operation performed after the index procedure during the available follow-up period.

Hiatal hernia recurrence was assessed through retrospective review of hospital and available primary-care records and was recorded when a recurrent hernia was explicitly documented following clinical assessment, investigation or reintervention. Patients without documented recurrence were classified as having no clinically recorded recurrence. Routine radiological, endoscopic or physiological surveillance was not performed.

### Statistical analysis

All statistical analyses were performed using R version 4.5.2. Total operative time was designated as the primary outcome; analyses of secondary outcomes were exploratory. Continuous variables were assessed for normality and are reported as median and interquartile range. Between-group comparisons were performed using the Wilcoxon rank-sum test. Categorical variables are presented as number and percentage and were compared using Fisher’s exact test or Pearson’s chi-squared test, as appropriate, with Fisher’s exact test used when expected cell counts were small. Clavien–Dindo complication categories were compared separately using two-sided Fisher’s exact tests. Absolute standardised mean differences were calculated for baseline characteristics to describe between-group balance independently of sample size. Effect estimates are presented with 95% confidence intervals where estimable.

For the highest-volume robotic surgeon, an exploratory sequential analysis was undertaken using study-period case number. The relationship between sequential case number and operative time was examined using simple linear regression, and a cumulative sum plot of operative-time deviations from the surgeon’s study-period mean was inspected descriptively. These analyses were not used to define a proficiency threshold. The principal sensitivity analysis excluded revisional procedures, producing primary-operation cohorts of 38 robotic and 38 laparoscopic cases. Within this cohort, operative time was log-transformed and analysed using multivariable linear regression with heteroscedasticity-consistent HC3 standard errors. The model included operative approach, calendar year, Nissen 360° fundoplication, age, BMI and ASA grade. Adjusted coefficients were exponentiated and reported as time ratios.

Same-day discharge was analysed in the primary-operation cohort using multivariable logistic regression, including operative approach, calendar year, Nissen 360° fundoplication, age, BMI and ASA grade. Adjusted effects were reported as odds ratios with 95% confidence intervals. Surgeon-level operative-time findings were reported descriptively because several surgeon-by-approach groups were sparse and highly imbalanced. A separate sensitivity analysis excluded the highest-volume robotic surgeon. Adjusted analyses of postoperative dysphagia, readmission and recurrence were not undertaken because the small number of events would have produced unstable estimates.

## Results

A total of 123 fundoplications were performed during the study period. Eighteen procedures performed in conjunction with Heller’s myotomy and five emergency cases were excluded, leaving 100 elective fundoplications for analysis. Of these, 57 were performed robotically and 43 laparoscopically. Baseline demographic characteristics of the robotic and laparoscopic cohorts are summarised in Table 1.

**Table 1:** Baseline demographic characteristics of the study cohort

Variable	Total (*n* = 100)	Laparoscopic (*n* = 43)	Robotic (*n* = 57)	*p* value
Age, median [IQR]	60 [49–70]	65 [52–72]	58 [48–69]	0.258
BMI, median [IQR]	28.2 [25.8–31.1]	27.4 [25.7–31.1]	28.6 [25.8–30.9]	0.508
Sex, n (%)				0.196
Female	68 (68%)	26 (60.5%)	42 (73.7%)	
Male	32 (32%)	17 (39.5%)	15 (26.3%)	
ASA class, n (%)				1.000
ASA 1	14 (14%)	6 (14.0%)	8 (14.0%)	
ASA 2	60 (60%)	26 (60.5%)	34 (59.6%)	
ASA 3	26 (26%)	11 (25.6%)	15 (26.3%)	

Baseline demographic characteristics of the study cohort. Continuous variables are reported as median [interquartile range] and categorical variables as n (%). Comparisons were performed using the Wilcoxon rank-sum test and Fisher’s exact test, as appropriate. Absolute standardised mean differences describe between-group balance independently of sample size, with values below 0.10 indicating minimal imbalance. BMI, body mass index; ASA, American Society of Anesthesiologists; SMD, standardised mean difference

### Indication for surgery

Indications for surgery between laparoscopic and robotic cohorts are shown below (Table 2). Hiatal hernia with reflux was the most common indication in both groups, accounting for 23 of 43 laparoscopic cases and 32 of 57 robotic cases. Revisional surgery, particularly for recurrent hiatal hernia, represented a greater proportion of robotic procedures compared with laparoscopic procedures (13 vs. 3 cases). Medically refractory reflux was more frequently managed laparoscopically (11 vs. 5 cases), while post-fundoplication dysphagia occurred at similar frequency in both cohorts.

**Table 2:** Indication for surgery

Indication	Total	Laparoscopic	Robotic
Anti-Reflux	16	11 (25.6%)	5 (8.8%)
Hiatal hernia	5	4 (9.3%)	1 (1.7%)
Hiatal hernia with reflux	55	23 (53.4%)	32 (56.1%)
Recurrent hiatal hernia	16	3 (7%)	13 (22.8%)
Recurrent Anti-reflux	3	0	3 (5.2%)
Post-Fundoplication Dysphagia	5	2 (4.7%)	3 (5.3%)

Distribution of surgical indications by operative approach. Values are reported as n (%)

### Revisional surgery

Revisional procedures were more commonly performed using a robotic approach, accounting for 19 of 57 robotic cases (33.3%) compared to only 5 of 43 laparoscopic cases (11.6%). Robotic surgery was associated with a higher likelihood of being selected for revisional cases (odds ratio 3.8, 95% CI 1.3–11.0; *p* = 0.006). Indications for revisional surgery included recurrent reflux, dysphagia following previous fundoplication, and failure of prior anti-reflux procedures.

### Operative time

Operative times for the laparoscopic and robotic approaches are shown in Table 3. Total operative time was defined as the interval from skin incision to skin closure. Docking time was defined as the interval from patient positioning to commencement of console operation, while console time was defined as the interval from initiation of console control to completion of console-based operative steps.

**Table 3:** Operative time metrics

Variable	Total	Laparoscopic	Robotic	*P* value
Total operative time, min (median [IQR])	100 [75–136.25]	120 [90–144]	88 [70–120]	0.017
Docking time, min (median [IQR])			6 [4–9]	
Console time, min (median [IQR])			64 [50–91]	

Operative time metrics by surgical approach. Operative times are reported as median [IQR], with between-group comparisons performed using the Wilcoxon rank-sum test

**Fig. 1:**
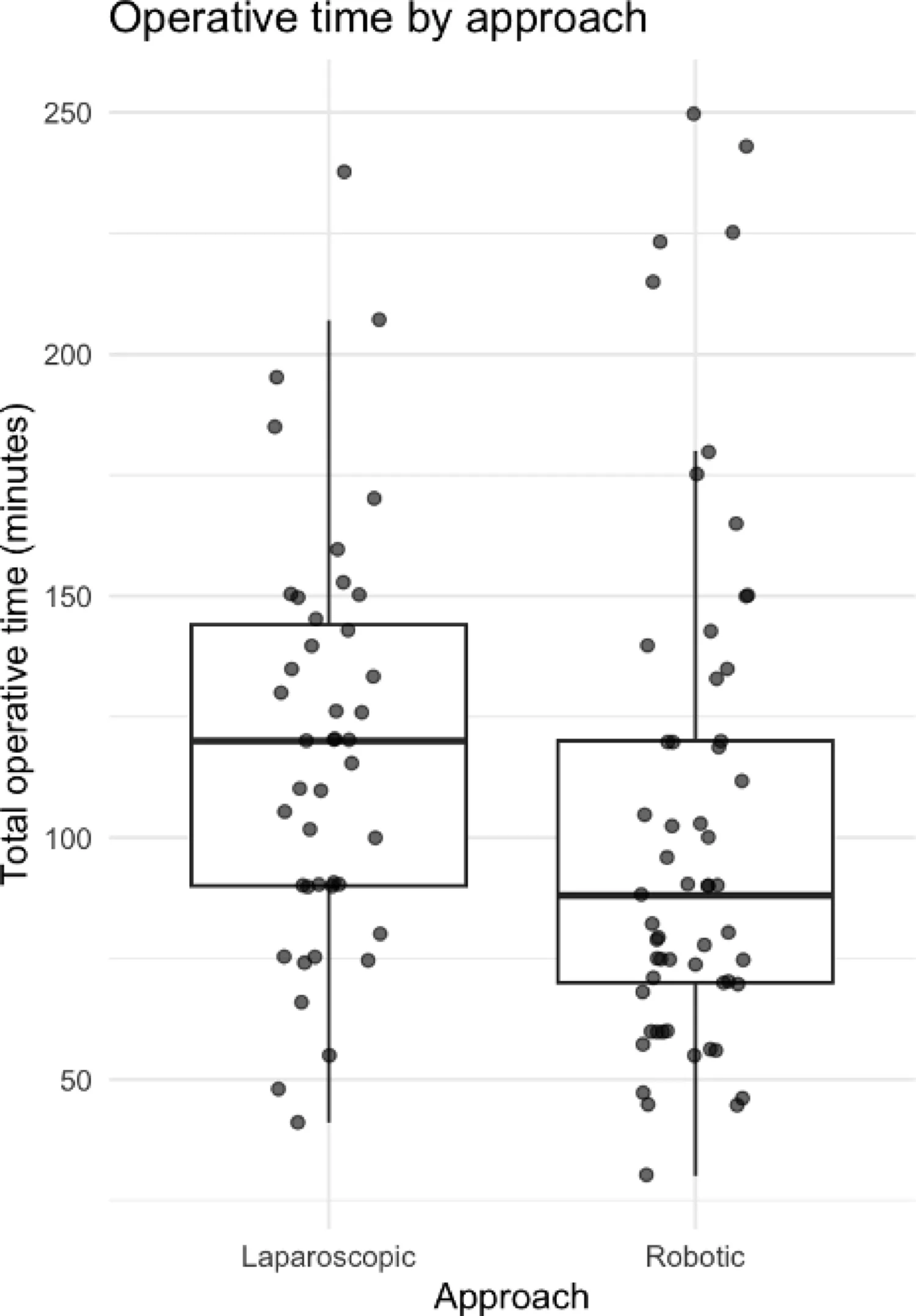
Operative time by surgical approach. Boxplot demonstrating total operative time (minutes) for laparoscopic and robotic fundoplication. Central lines represent the median

Median total operative time was 120 min [IQR 90–144] for laparoscopic procedures and 88 min [IQR 70–120] for robotic procedures, representing a difference in medians of 32 min (*W* = 1568; *p* = 0.017) as shown in **Figure 1**. Among robotic cases, median docking time was 6 min [IQR 4–9] and median console time was 64 min [IQR 50–91]. 

The highest-volume robotic surgeon was involved in 35 of 57 robotic procedures (61.4%). Excluding all procedures involving this surgeon left 22 robotic cases, with a median operative time of 93 min [IQR 62.5–153.8], compared with 120 min [IQR 90–144] in the laparoscopic cohort (*p* = 0.255). Sequential analysis of the highest-volume surgeon’s 35 robotic cases found no significant association between case number and operative time (+ 0.71 min per case, 95% CI − 0.82 to 2.24; *p* = 0.350; R²=0.027). Exploratory CUSUM analysis identified no sustained change point (See Supp. Table S2). These findings suggest that the observed operative-time pattern was not explained by a measurable learning trend during the study period.

Nine laparoscopic procedures (20.9%) were performed by trainees under direct consultant supervision. These procedures were distributed across the operative-time range, and their exclusion did not alter the median laparoscopic operative time. No robotic procedures were performed by trainees during the study period.

After excluding revisional procedures, 38 primary operations remained in each group. Median operative time was 77 min [IQR 62–118] robotically and 120 min [IQR 90–144.5] laparoscopically (*p* < 0.001). Median length of stay was 0 days [IQR 0–1] following robotic surgery and 1 day [IQR 1–2] following laparoscopic surgery (*p* = 0.003). Same-day discharge occurred in 23/38 robotic cases (60.5%) and 9/38 laparoscopic cases (23.7%; OR 4.94, 95% CI 1.83–13.31; *p* = 0.002).

Operative approach varied substantially by calendar period, with laparoscopic surgery predominating in 2022 and robotic utilisation increasing from three cases in 2022 to 29 in 2025; robotic same-day discharge reached 72.4% in 2025. Calendar year was therefore included alongside Nissen 360° fundoplication, age, BMI and ASA grade in the adjusted analyses (See Supp. Tables 4–6). The adjusted robotic operative-time ratio was 0.89 (95% CI 0.72–1.09; *p* = 0.250), while the adjusted odds of same-day discharge remained higher following robotic surgery (OR 5.02, 95% CI 1.09–23.00; *p* = 0.038). Annual operative-time and discharge patterns are presented in Supplementary Table S5.

**Table 4:** Intra-operative hiatal repair and fundoplication wrap type

	Technique	Laparoscopic	Robotic	Total
**Cruroplasty**	Anterior	1 (2.3%)	1 (1.8%)	2 (2%)
	Posterior	15 (34.9%)	20 (35.1%)	35 (35%)
	Both	27 (62.8%)	31 (54.4%)	58 (58%)
	None		5 (8.8%)	5 (5%)
**Wrap type**	Anterior 180°	25 (58.1%)	10 (17.5%)	35 (35%)
	No wrap		1 (1.8%)	1 (1%)
	Nissen 360°	16 (37.2%)	44 (77.2%)	60 (60%)
	Posterior 180°	2 (4.7%)	2 (3.5%)	4 (4%)

Hiatal repair and fundoplication techniques are presented by surgical approach. Data are reported as number (percentage) within each approach. “Both” refers to combined anterior and posterior cruroplasty. Wrap configurations include anterior 180°, no wrap, posterior 360° (Nissen), and posterior 180° fundoplications

**Table 5:** Primary-surgery sensitivity analysis and adjusted perioperative outcomes

Outcome	Laparoscopic *n* = 38	Robotic *n* = 38	Effect estimate	*p* value
Operative time, median [IQR]	120 [90–144.5] min	77 [62–118] min	Median difference − 43 min	< 0.001
Length of stay, median [IQR]	1 [1–2] days	0 [0–1] days	Median difference − 1 day	0.003
Same-day discharge	9/38 (23.7%)	23/38 (60.5%)	OR 4.94 95% CI 1.83–13.31	0.002
Adjusted operative time	1.00	Ratio 0.89	95% CI 0.72–1.09	0.250
Adjusted same-day discharge	1.00	OR 5.02	95% CI 1.09–23.00	0.038

Data are median (IQR) or n/N (%). Adjusted models included calendar year, Nissen 360° fundoplication, age, BMI and ASA grade, with laparoscopy as the reference. CI, confidence interval; OR, odds ratio

### Intra-operative technique

All procedures were completed using a minimally invasive approach, consistent with ARROW audit criteria [2]. Intraoperative techniques are shown in Table 4. Combined anterior and posterior cruroplasty was the most frequently used hiatal repair technique, performed in 58 patients (58%), including 62.8% of laparoscopic and 54.4% of robotic cases. Posterior-only cruroplasty was performed in 35 patients (35%), while isolated anterior repair was uncommon (2%). Five patients underwent no cruroplasty.

Nissen 360° fundoplication was the most common wrap overall (60%) and was used more frequently in robotic than laparoscopic procedures (77.2% versus 37.2%). Conversely, anterior 180° fundoplication was more frequent laparoscopically (58.1% versus 17.5%). Posterior 180° fundoplication and procedures without wrap formation were uncommon. Gastropexy was performed in six patients (6%).

The difference in wrap configuration reflected surgeon preference, operative indication and case selection rather than a platform-mandated technique. Hiatal hernia type and anatomical complexity may also have influenced case allocation, as type I hernias are not directly comparable with type II–IV hernias. The limited numbers within individual hernia categories prevented reliable stratified analysis.

### Post-operative outcomes

Postoperative outcomes are summarised in Table 5. Median length of stay was shorter following robotic surgery than laparoscopy (0 days [IQR 0–1] versus 1 day [IQR 0–2]; *p* = 0.001). Same-day discharge was achieved in 36/57 robotic cases (63.2%) and 12/43 laparoscopic cases (27.9%), giving an overall rate of 48%. The unadjusted odds of same-day discharge were higher following robotic surgery (OR 4.43, 95% CI 1.88–10.43; *p* < 0.001).

**Table 6:** Post-operative outcomes

Outcome	Laparoscopic *n*/43 (%)	Robotic *n*/57 (%)	Total *n*/100 (%)	*p* value
Length of stay (days), median (IQR)	1 (2)	0 (1)	1 (1)	**0.001**
Day case rate n (%)	12 (27.9%)	36 (63.2%)	48 (48%)	**0.0006**
Complications (Clavien–Dindo), n (%)				0.281
Nil	33 (76.7%)	39 (68.4%)	72 (72%)	0.38
CD I–II	4 (9.3%)	12 (21.1%)	16 (16%)	0.17
CD III	5 (11.6%)	6 (10.5%)	11 (11%)	1.00
CD IV	0 (0%)	0 (0%)	0 (0%)	-
CD V	1 (2.3%)	0 (0%)	1 (1%)	0.43
Return to theatre (30 days), n (%)	1 (2.3%)	2 (3.5%)	3 (3%)	1.000
Redo fundoplication, n (%)	4 (9.3%)	4 (7%)	8 (8%)	0.722
30-day readmission, n (%)	1 (2.3%)	7 (12.3%)	8 (8%)	0.133
Hiatal hernia recurrence†, n/N (%)	4/30 (13.3%)	2/46 (4.3%)	6/76 (7.9%)	0.191

Continuous variables are reported as median (interquartile range) and compared using the Wilcoxon rank-sum test due to non-normal distribution. Categorical outcomes are presented as number (%) and were compared using Fisher’s exact test for low-frequency events and Pearson’s chi-squared test otherwise, as appropriate. †Hiatal hernia recurrence was assessed only in patients with an index hiatal hernia; cases without a hernia were excluded from the denominator

Most patients were managed postoperatively with routine surgical ward care without higher-level organ support. One highly comorbid patient in the laparoscopic cohort required unplanned intensive care admission and subsequently died following postoperative complications. This event is reported descriptively, and no formal adjudication of causality was undertaken.

Complications are reported separately by operative approach and Clavien–Dindo grade in Table 6 . In the laparoscopic cohort, four patients (9.3%) experienced grade I–II complications, five (11.6%) experienced grade III complications and one (2.3%) experienced a grade V complication. In the robotic cohort, 12 patients (21.1%) experienced grade I–II complications and six (10.5%) experienced grade III complications. No grade IV complications occurred. The small number of major events precluded conclusions regarding equivalent safety.

Return to theatre within 30 days occurred in three patients (3%), and eight patients (8%) underwent subsequent redo fundoplication during the available follow-up. Thirty-day readmission occurred in 7/57 robotic cases and 1/43 laparoscopic cases (OR 5.88, 95% CI 0.70–49.73; *p* = 0.133). Among the seven robotic readmissions, three involved postoperative dysphagia, three involved chest or epigastric pain or nausea with reassuring investigation, and one was treated for a chest infection. One patient with dysphagia required return to theatre for conversion to an anterior fundoplication. The single laparoscopic readmission involved endoscopic treatment for postoperative dysphagia. The wide confidence interval and small number of readmissions limit the precision of this estimate and preclude attribution of the difference to operative approach.

### Primary-surgery sensitivity and adjusted analyses

After excluding revisional procedures, 38 primary operations remained in each group. Median operative time was 77 min (IQR 62–118) robotically and 120 min (IQR 90–144.5) laparoscopically, representing an unadjusted difference in medians of 43 min (*p* < 0.001). Median length of stay was 0 days (IQR 0–1) after robotic surgery and 1 day (IQR 1–2) after laparoscopic surgery (*p* = 0.003). Same-day discharge occurred in 23/38 robotic cases (60.5%) and 9/38 laparoscopic cases (23.7%), corresponding to an unadjusted OR of 4.94 (95% CI 1.83–13.31; *p* = 0.002).

After adjustment for calendar year, Nissen 360° fundoplication, age, BMI and ASA grade, the robotic operative-time ratio was 0.89 (95% CI 0.72–1.09; *p* = 0.250), equivalent to an estimated 11% shorter operative time, although the confidence interval included no difference. The association with same-day discharge remained statistically significant after adjustment (OR 5.02, 95% CI 1.09–23.00; *p* = 0.038), although the wide confidence interval indicates limited precision.

### Postoperative instructions and follow-up

Postoperative dietary advice was documented for all patients (Table 7). At the latest documented review, PPI therapy was recorded as discontinued in 63% of total cases. PPI status was considered a clinically relevant postoperative outcome but not an objective measure of reflux control.

**Table 7:** Post-operative instructions and follow-up

Outcome	Result
Dietary advice documented n (%)	100 (100%)
PPI continued at latest documented review n (%)	Yes: 37 (37%) No: 63 (63%)
Discharged from surgical follow-up n (%)	Yes: 71 (71%) No: 29 (29%)
Follow-up provider n (%)	UGI CNS: 28 (28%) UGI surgeon: 20 (20%) Both: 48 (48%) None documented: 4 (4%)
Follow-up duration, months, median [IQR]	Overall: 3 [2–6] Laparoscopic: 4 [2–9] Robotic: 2 [2–5] *p* = 0.082

Data are presented as number (percentage) unless otherwise stated. Follow-up duration is presented as median [IQR] and was compared between approaches using the Wilcoxon rank-sum test. PPI, proton pump inhibitor; CNS, clinical nurse specialist

Seventy-one patients (71%) were discharged from routine surgical follow-up after postoperative review. Follow-up was provided by both the UGI surgeon and clinical nurse specialist in 48%, the clinical nurse specialist alone in 28%, and the UGI surgeon alone in 20%; 4% had no documented follow-up. Median documented follow-up was 3 months overall, comprising 4 months [IQR 7] following laparoscopic surgery and 2 months [IQR 3] following robotic surgery (*p* = 0.082).

Although median scheduled follow-up was short, 76 patients had at least 12 months between surgery and data collection and underwent review of available hospital and GP records. This record-based review could identify clinically documented recurrence, subsequent investigation or reintervention but did not include routine radiological, endoscopic or physiological surveillance.

## Discussion

This study demonstrates that robotic anti-reflux and hiatal surgery can be integrated successfully into an established secondary-care benign UGI service, including for patients referred from neighbouring centres and a substantial revisional workload. The robotic programme achieved favourable operative times, length of stay and same-day discharge. However, these findings reflect the performance of the programme as a whole and should not be interpreted as isolated effects of the robotic platform.

Robotic procedures had shorter median operative times than laparoscopic procedures despite a greater revisional workload (88 versus 120 min; *p* = 0.017). The direction of this difference persisted in the primary-only and surgeon-restricted analyses, although adjustment for calendar year, Nissen 360° fundoplication, age, BMI and ASA grade attenuated the association to an estimated 11% reduction that was not statistically significant. Exclusion of the highest-volume robotic surgeon similarly produced a non-significant difference, although robotic procedures remained 27 min shorter. Sequential and CUSUM analyses of this surgeon’s 35 robotic cases identified no measurable learning trend. Notably, the surgeon began most of their robotic practice during the study period, but had many previous years of open and laparoscopic UGI experience, suggesting that established procedure-specific expertise may have transferred effectively to the robotic platform. However, robotic experience was also acquired through other procedures. The findings therefore demonstrate efficient robotic delivery within an experienced UGI programme, although surgeon expertise, case allocation and platform effects remain inseparable.

The technical characteristics of robotic surgery provide a plausible explanation for this efficiency signal. Three-dimensional magnified vision, wristed instrumentation and stable exposure may facilitate dissection, crural repair and intracorporeal suturing, particularly in revisional surgery involving fibrosis and distorted anatomy. Similar technical advantages have been described in robotic Heller myotomy, where precise dissection around the oesophagogastric junction is especially important [13]. Improved precision and surgeon ergonomics are valuable technical features, but do not necessarily translate into superior patient-level outcomes.

Treatment era and operative technique provide important additional context. Robotic utilisation increased from three cases in 2022 to 29 in 2025 alongside maturation of the programme and its perioperative pathways. Calendar year was therefore incorporated into the adjusted analyses. Wrap configuration also differed substantially, with Nissen 360° fundoplication predominating robotically and anterior 180° fundoplication more common laparoscopically. This reflected surgeon preference, indication and case selection rather than a platform requirement. Hernia type and anatomical complexity may have contributed further. The limited numbers within individual categories prevented reliable stratified analysis.

Robotics was associated with shorter length of stay and higher day-case utilisation, and the association with same-day discharge remained significant after adjustment. The robotic day-case rate of 63.2% compares favourably with national laparoscopic rates of 7.4–15.1% [14] and builds upon previously reported day-case experience within this unit [15]. However, same-day discharge is determined by the wider perioperative pathway and cannot be attributed solely to operative platform. Most robotic procedures were performed by surgeons who were established advocates of day-case surgery, whereas the laparoscopic cohort included lower-volume surgeons with differing discharge practices. This introduces residual confounding by surgeon preference, patient selection and intended postoperative pathway. Planned day-case status, operating-list position, anaesthetic pathway and changes in formal discharge criteria were not consistently recorded and could not be incorporated into the model. The finding should therefore be interpreted as evidence that robotic surgery was successfully integrated into an institutional day-case pathway. The finding therefore demonstrates successful integration of robotics into an established day-case pathway rather than an intrinsic platform effect.

Early postoperative outcomes were broadly reassuring. Most patients experienced no complication or only a Clavien–Dindo grade I–II event. Grade III complications occurred in 11.6% of laparoscopic and 10.5% of robotic cases, no grade IV complications occurred, and return to theatre was uncommon. Published experience has similarly demonstrated the feasibility of robotic anti-reflux surgery with acceptable short-term outcomes [4, 16]. Major complication rates did not differ significantly between approaches, providing reassuring early safety data, although the small number of events precludes formal conclusions of equivalence.

The overall 30-day readmission rate was 8%, remaining within the national benchmark of less than 10% [2]. Readmission was numerically higher in the robotic cohort (12.3% vs. 2.3%), although this did not reach statistical significance. Interpretation is limited by the small absolute number of events, with only eight readmissions across the cohort, reducing statistical power for rare postoperative outcomes. The reasons for readmission included dysphagia, pain management, nausea and chest infection. Several patients had reassuring imaging or endoscopic findings and were managed conservatively. Most readmissions were managed conservatively, reflected by the low overall reoperation rate of 3% at 30 days, again within national benchmark of < 5% [2, 8]. Potential contributors include the greater revisional workload, more frequent Nissen fundoplication, higher same-day discharge and a lower threshold for reassessment within the developing robotic pathway. These remain hypotheses, and a clinically important difference cannot be excluded from only eight events.

Postoperative follow-up and recovery patterns were analysed. All patients received documented dietary guidance following surgery and were discharged once tolerating oral fluid intake, progressing from liquids to soft diet as per standard post-fundoplication protocols. The majority of patients (63%) discontinued proton pump inhibitor therapy after surgery, comparing favourably with published literature reporting PPI cessation rates of 40.5% in a series of over 22,000 patients [17]. Medication independence is a clinically relevant and commonly reported outcome after anti-reflux surgery, but PPI use is influenced by prescribing practice, patient preference and alternative indications for acid suppression. Validated postoperative patient-reported outcome measures were not routinely collected, limiting assessment of symptom control.

Median scheduled follow-up was was 3 months across both groups. During the study period, available GP records were reviewed for 76 patients with at least 12 months between surgery and data collection, allowing identification of clinically documented recurrence, subsequent investigation or reintervention. However, surveillance was not routinely supported by radiological, endoscopic or physiological assessment. The recurrence findings therefore represent early clinically recorded events and cannot establish comparative anatomical durability or exclude asymptomatic recurrence. Follow-up was predominantly shared between UGI surgeons and clinical nurse specialists, reflecting an integrated multidisciplinary pathway.

Formal economic conclusions are beyond the scope of this study. Robotic platforms carry substantial acquisition, maintenance, instrument, training and amortisation costs [15]. Shorter operative times, greater day-case utilisation, reduced length of stay and avoidance of advanced disposable energy devices may have favourable resource implications, particularly where robotic infrastructure is already shared across specialties. Whether these efficiencies offset the full costs of programme delivery and downstream healthcare use requires prospective health-economic evaluation.

### Limitations

These findings should be interpreted within the context of a retrospective, non-randomised, single-centre study. The robotic cohort included more revisional and complex procedures, and operative approach was influenced by case allocation, surgeon and treatment era. Primary-only, adjusted and sensitivity analyses were therefore undertaken to explore these differences, although some residual confounding is unavoidable and the contribution of the platform cannot be fully separated from that of the wider programme. Intended day-case status, list position and individual discharge practices were not consistently recorded; therefore, the same-day discharge findings are best interpreted as reflecting the robotic programme and its associated pathway.

The sample size was sufficient to describe programme implementation and common perioperative outcomes but provided less precision for uncommon events such as major complications, readmission, reoperation and recurrence. Follow-up primarily captured early clinical outcomes, without standardised symptom scoring, physiological testing or routine anatomical surveillance. Formal health-economic evaluation was beyond the study scope, and wider applicability should be assessed in other benign UGI services. Finally, the analyses were exploratory, and the possibility of type I error from multiple comparisons should be acknowledged.

## Conclusion

Robotic anti-reflux and hiatal surgery was successfully integrated into an established benign upper gastrointestinal service, including referrals from neighbouring trusts and a substantial revisional workload, without an observed deterioration in early perioperative outcomes. Although operative duration has traditionally been considered a limitation of robotic surgery, the favourable operative times in this series demonstrate its potential for efficient service delivery. Laparoscopic fundoplication continued to provide reliable outcomes, reinforcing its role as the established standard and showing how both approaches can support tailored care in routine NHS practice. The relative contributions of platform, surgeon expertise, case selection and treatment era require evaluation in larger prospective studies with longer follow-up and formal economic assessment.

## Supplementary Information

Below is the link to the electronic supplementary material.

Supplementary Material 1

## Data Availability

No datasets were generated or analysed during the current study.
