# Supplementary material for: Robotic versus laparoscopic anti-reflux and hiatal surgery in a uk benign upper gi unit: perioperative outcomes in a four-year observational study
Source: J Robot Surg. 2026 Sep 21;20(1):1010. doi: 10.1007/s11701-026-03988-0 (PMC13593650; doi:10.1007/s11701-026-03988-0)
Supplement: Supplementary file 1 [file 11701_2026_3988_MOESM1_ESM.docx]

### **Supplementary Data**

**ARROW Audit Criteria**

The current study utilised benchmark standards derived from national and international guidelines to evaluate adherence to best practice in the perioperative assessment and outcomes of anti-reflux surgery. These standards are summarised as follows:

- Esophageal manometry is mandatory in the preoperative work-up for anti-reflux surgery, with documentation required in the patient record (BSG and ICARUS guidelines) [9-10] – Expectation: 100%.
- Reflux monitoring is mandatory in patients with non-erosive gastroesophageal reflux disease (GERD), as per ICARUS guidelines, with documentation in the patient record [10] – Expectation: 100%.
- Gastroscopy must be performed within one year prior to anti-reflux surgery, in accordance with ICARUS guidelines [10] – Expectation: 100%.
- Laparoscopic approach is expected for anti-reflux surgery, with <5% open conversion rate at the unit level, based on AUGIS service provision guidance [8] – Expectation: 95%.
- Unplanned readmission should occur in less than 10% of patients at 30 days postoperatively, as per AUGIS standards [8] – Expectation: 90%.
- Unplanned reoperation should occur in fewer than 5% of cases at 30 days, according to AUGIS guidelines [8] – Expectation: 95%.

**Preoperative Investigations**

Pre-operative investigations are summarised in Table 3. Gastroscopy was performed in 82% of patients. Oesophageal manometry and pH testing were completed in approximately half of the cohort (53% and 54%, respectively), with a small number of studies attempted but unsuccessful. Barium swallow was performed in 34% overall and was frequently used when physiological testing was not performed or could not be completed. Among patients without manometry, 15 underwent barium swallow, and all unsuccessful manometry or pH studies were followed by contrast imaging. In addition, 11 of 18 patients without pre-operative gastroscopy had a barium swallow performed.

trend during the study period. Previous surgical experience and changing case complexity were not fully captured.

**Operating Time**

Operative time was assessed across the highest-volume robotic surgeon’s 35 chronologically ordered cases. There was no significant association between sequential case number and operative time (+0.71 minutes per case, 95% CI −0.82 to +2.24; p = 0.350; R² = 0.027). Exploratory CUSUM analysis showed no clear sustained change point, suggesting that the operative-time pattern was not explained by a measurable learning Four surgeons performed cases using both approaches, but within-surgeon comparisons were limited by marked imbalance in approach-specific case numbers. Only one surgeon performed more than five operations using each approach. Surgeon-specific results were therefore reported descriptively without formal inferential comparison.

After excluding the highest-volume robotic surgeon, the robotic median operative time remained numerically shorter than the laparoscopic median (93 vs 120 minutes), but the difference was not statistically significant (p = 0.255). This suggests that the unadjusted operative-time difference may reflect a combination of platform, surgeon experience, case selection and treatment era.

Operative approach was strongly associated with calendar period. Laparoscopic surgery predominated in 2022, whereas 29 of 36 operations performed in 2025 were robotic. Same-day discharge also increased within the robotic programme over time. Calendar year was therefore included in the adjusted analyses to account partially for treatment-era effects.

Robotic utilisation increased during the study period, from 3 cases in 2022 to 29 cases in 2025, while robotic same-day discharge reached 72.4% in 2025. Operative-time and discharge patterns by calendar year are presented descriptively in Supplementary Table S5.

## Supplementary Table S1. Pre-operative investigations

| **Investigation** | **Performed** | **Attempted** | **Not Performed** |
| --- | --- | --- | --- |
| **Gastroscopy (OGD)** | 82 (82%) | — | 18 (18%) |
| **Manometry** | 53 (53%) | 5 (5%) | 42 (42%) |
| **pH testing** | 54 (54%) | 2 (2%) | 44 (44%) |
| **Barium swallow** | 34 (34%) | — | 66 (66%) |

Pre-operative investigations are presented as n (%) for the overall cohort.

## Supplementary Table S2. Sequential robotic operative-time analysis

| **Analysis** | **Estimate** | **95% CI** | **p value** |
| --- | --- | --- | --- |
| Change in operative time per additional case | +0.71 minutes | −0.82 to +2.24 | 0.350 |

## Supplementary Table S3: Robotic Qualified Surgeons Case Numbers and Operative Time

| **Surgeon** | **Laparoscopic n** | **Laparoscopic median [IQR], min** | **Robotic n** | **Robotic  median [IQR], min** |
| --- | --- | --- | --- | --- |
| Surgeon A | 3 | 48 [45–117] | 34 | 85 [70–120] |
| Surgeon B | 1 | 55 [55–55] | 14 | 75 [58–161] |
| Surgeon C | 7 | 153 [125–178] | 6 | 104 [83–152] |
| Surgeon D | 1 | 115 [115–115] | 2 | 105 [98–113] |

## Supplementary Table S4 : Operative time after exclusion of highest volume surgeon

| **Approach** | **Cases n** | **Operative time (mins) median [IQR]** | **p value** |
| --- | --- | --- | --- |
| Laparoscopic | 43 | 120 [90–144] | 0.255 |
| Robotic after exclusion | 22 | 93 [63–154] |  |

## Supplementary Table S5: Distribution of operative approach, operative time and same-day discharge by calendar year.

| **Year** | **Laparoscopic operative time**  **n**  **median [IQR]** | **Robotic operative time**  **n**  **median [IQR]** | **Laparoscopic same-day discharge** | **Robotic same-day discharge** |
| --- | --- | --- | --- | --- |
| 2022 | n = 17  130 [107–160] | n = 3  60 [54–80] | 4/17  (23.5%) | 3/3  (100%) |
| 2023 | n = 10  110 [93.8–132.5] | n = 11  119 [90–180] | 4/10  (40.0%) | 5/11  (45.5%) |
| 2024 | n = 9  115 [90–130] | n = 14  95 [70–147.5] | 3/9  (33.3%) | 7/14  (50.0%) |
| 2025 | n = 7  102 [63–141] | n = 29  79 [64–105] | 1/7  (14.3%) | 21/29  (72.4%) |
